# Prognostic value of PD-L1 expression in patients with unresectable stage III non-small cell lung cancer treated with chemoradiotherapy

**DOI:** 10.1186/s13014-020-01696-z

**Published:** 2020-10-29

**Authors:** Martina Vrankar, Izidor Kern, Karmen Stanic

**Affiliations:** 1 Department of Radiotherapy, Institute of Oncology Ljubljana, Zaloska 2, 1000 Ljubljana, Slovenia; 2 Department of Pathology, University Clinic of Respiratory and Allergic Diseases Golnik, Golnik 36, 4202 Golnik, Slovenia; 3 Faculty of Medicine, University of Ljubljana, Vrazov trg 2, 1000 Ljubljana, Slovenia

**Keywords:** Non small cell lung cancer, PD-L1 expression, Stage III, Chemoradiotherapy

## Abstract

**Background:**

Expression of PD-L1 is the most investigated predictor of benefit from immune checkpoint blockade in advanced NSCLC but little is known about the association of PD-L1 expression and clinicopathological parameters of patients with unresectable stage III NSCLC.

**Methods:**

National registry data was searched for medical records of consecutive inoperable stage III NSCLC patients treated with ChT and RT from January 2012 to December 2017. Totally 249 patients were identified that met inclusion criteria and of those 117 patients had sufficient tissue for PD-L1 immunohistochemical staining.

**Results:**

Eighty patients (68.4%) expressed PD-L1 of ≥ 1% and 29.9% of more than 50%. Median PFS was 15.9 months in PD-L1 negative patients and 16.1 months in patients with PD-L1 expression ≥ 1% (*p* = 0.696). Median OS in PD-L1 negative patients was 29.9 months compared to 28.5 months in patients with PD-L1 expression ≥ % (*p* = 0.888). There was no difference in median OS in patients with high PD-L1 expression (≥ 50%) with 29.8 months compared to 29.9 months in those with low (1–49%) or no PD-L1 expression (p = 0.694). We found that patients who received a total dose of 60 Gy or more had significantly better median OS (32 months vs. 17.5 months, p < 0.001) as well as patients with PS 0 (33.2 vs. 20.3 months, p = 0.005).

**Conclusions:**

In our patients PD-L1 expression had no prognostic value regarding PFS and OS. Patients with good performance status and those who received a total radiation dose of more than 60 Gy had significantly better mOS.

## Introduction

For decades, treatment of patients with surgically unresectable, locally advanced non-small cell lung cancer (NSCLC) had revealed no substantial progress. Recently, the results of the Pacific trial of maintenance therapy with Programmed Death Ligand 1 (PD-L1) antibody durvalumab in patients without progression after concurrent chemoradiotherapy have revolutionized the management of patients and opened great interest in new developing field of research in stage III NSCLC [1–3]. PD-L1 pathway is one of the most important mechanisms for tumor cells to escape the immunosurveillance of tumor-infiltrating lymphocytes (TILs) in NSCLC [4, 5]. Expression of PD-L1 on tumor cells is one of the most investigated predictors of benefit from immune checkpoint blockade in advanced NSCLC but little is known about the association of PD-L1 expression and clinicopathological parameters including prognosis of patients with unresectable stage III NSCLC [6–10]. In our previous research, we assessed the impact of PD-L1 expression on tumor cells from patients with unresectable stage III NSCLC who were treated in our institution between 2005 and 2010 in prospective trial with concurrent chemotherapy (ChT) and radiotherapy (RT) [11]. We found significant shorter progression free survival (PFS) and overall survival (OS) in patients with PD-L1 expression. Since only small number of patients were evaluable, the findings were not conclusive. However, the prognostic value of PD-L1 expression is still unclear with some suggesting data that is more frequently detected in higher stages [12, 13]. In present retrospective analysis, we investigated the clinical importance of PD-L1 expression in consecutive patients with unresectable stage III NSCLC treated with RT and ChT in our institution from 2012 to 2017 and correlated their expression with clinical characteristics, including patients’ outcomes. In addition, since systemic inflammatory response to cancer has been described as a poor prognostic factor in NSCLC, we correlated the serum C-reactive protein (CRP) level and neutrophil to lymphocyte ratio (NLR) as most investigated inflammatory markers with PFS and OS [14, 15].

## Methods

### Patients

National registry data was searched for medical records of consecutive inoperable stage III (according to the Staging Manual in Thoracic Oncology, version 8, of the International Association for the Study of Lung Cancer [16]) NSCLC patients treated with ChT and RT with radical intent from January 2012 to December 2017. Inclusion criteria for retrospective analysis were three-dimensional computed tomography (CT)—based conformal RT with a linear accelerator photon beam of 5–10 MV with a minimum total dose of 54 Gy or more in 2 Gy fractions 5 times weekly. All included patients were treated with 1–5 cycles of induction and/or concomitant cisplatin based ChT. Totally 249 patients were identified that met inclusion criteria for tumor sample investigation and of those 117 patients had sufficient tissue for immunohistochemical staining.

### Tumor tissue

Archived tumor tissue of small biopsy samples were collected from patients with histologically confirmed diagnosis of NSCLC in a diagnostic work-up before any tumor directed treatment. At least 100 viable tumor cells was regarded as a sufficient tumor content to perform immunohistochemistry (IHC). The specimens were formalin-fixed (6–48 h), paraffin embedded (FFPE), freshly cut into 4 µm sections and stained for PD-L1 with a rabbit monoclonal antibody SP263 as part of the Ventana PD-L1 SP263 assay (Ventana/Roche, USA) on an automated platform (Benchmark, Ventana/Roche, USA). An OptiView DAB Detection Kit with Amplification Kit (Ventana, Roche, USA) was used according to the manufacturer’s instructions for visualization. Percentage of PD-L1 positive immunohistochemical reaction was evaluated in tumor cells, ranging from 0 to 100%. Tumor cells with circumferential or partial membranous staining of any intensity were considered positive. Staining threshold for PD-L1 positivity was set at 1% or higher in tumor cells. Human placenta and tonsil served as a positive control tissue for PD-L1 expression.

### Statistical analysis

Baseline characteristics of patients, including age, gender, histologic type, smoking status, CRP before and after treatment, neutrophil and lymphocyte levels before and after treatment, tumor node metastases (TNM) stage, and dates of progression were collected retrospectively from medical records. The NLR was calculated as neutrophil-to-lymphocyte ratio and the cut-off in our analysis was 5. All patients were reclassified according to 8^th^ TNM classification. Dates of diagnosis and death were obtained from National registry data.

Patients information that were assessed according to PD-L1 expression were patient demographics, pathological features, TNM stage, Eastern Cooperative Oncology Group performance status (ECOG PS), completed treatment, PFS and OS. The association between the PD-L1 expression and the clinicopathological variables of patients were tested using the Pearson’s chi-squared test or Fisher’s exact test. OS and PFS curves were estimated using Kaplan–Meier method. PFS and OS of patients were compared using the log-rank test. The Cox proportional hazards model was used to assess the association between OS, PD-L1 status and treatment characteristics. Results with values of p < 0.2 in univariate analysis were calculated in multivariate analysis. All tests were two tailed. A p value less than 0.05 was considered statistically significant. All p values reported were based on the two-sided hypothesis. The statistical analyses were calculated using SPSS-21 (IBM Corporation, Armonk, NY, USA).

PFS was defined as the time from the beginning of treatment to disease progression or death. OS was calculated as the time from the start of the treatment to death from any cause. Censoring was defined as the time from the beginning of treatment to the last contact with the patient and for alive patients, as the time from the beginning of treatment to the end of follow-up (January 2020).

## Results

Seven hundred sixty seven patients with unresectable NSCLC stage III were treated in our institution between January 2012 to December 2017. During that period 249 (32.5%) had combined sequential or concomitant chemo radiotherapy, 147 (19.2%) were treated with radical radiotherapy only and 371 (48.3%) had palliative radiotherapy. However, in 2017 palliative radiotherapy was reduced to 35.4% in favour of radical combined chemo radiotherapy in 45.6% of patients. The percentage of patients treated with radical radiotherapy only were similar, 19%.

Altogether, 117 patients of 249 treated with combined chemo radiotherapy had available tissue for PD-L1 IHC testing and were included in current analysis, of whom 80 (68.4%) expressed PD-L1 positivity of ≥ 1%. Median age of all patients was 61 years and the majority were male (67.5%), current or former smokers (96.5%). The most common histologic type was squamous cell carcinoma (60.7%) followed by adenocarcinoma (33.3%) and most patients had stage IIIB NSCLC (64.1%). Basic characteristics of patients are presented in Table 1. There were no significant differences between groups according PD-L1 expression and age, gender, histology, smoking status, TNM stage and ECOG PS. Considering the treatment completion, median biologically equivalent dose (EQD2) of RT was 60 Gy and median number of ChT cycles was 3 in both groups. The median follow-up was 45.3 months.

**Table 1:** Clinical characteristic of patients regarding PD-L1 expression

Characteristics	All, n	PD-L1 ≥ 1%, n	(%)	PD-L1 negative, n	(%)	p
All patients	117	80	68.4	37	31.6	
Age (years)						0.106
Median	61	62		60		
Range	47–77	47–77		50–69		
Gender						0.392
Female	38	28	35	10	27	
Male	79	52	65	27	73	
Histology						0.820
Squamous ca	71	47	58.8	24	64.9	
Adenoca	39	28	35	11	29.7	
Other	7	5	6.2	2	5.4	
Smoking status						0.829
Current	67	44	56.4	23	62.2	
Former	44	31	39.8	13	35.1	
Never	4	3	3.8	1	2.7	
Stage						0.079
IIIA	30	25	31.3	5	13.5	
IIIB	75	46	57.5	29	78.4	
IIIC	12	9	11.3	3	8.1	
ECOG PS						0.806
0	58	39	48.7	19	51.4	
1	57	40	50	17	45.9	
2	2	1	1.3	1	2.7	
RT dose (Gy)						0.793
Median		60		60		
Range		54–66		55–66		
ChT (number of cycles)						0.406
Median		3		3		
Range		1–5		1–5		
CRP after ChT/RT						0.242
≤ 2 × ULN	74	53	67.9	21	56.8	
> 2 × ULN	41	25	32.1	16	43.2	

*ChT* chemotherapy, *ECOG PS* Eastern Cooperative Oncology Group performance status, *n* number, *RT* radiotherapy, *CRP* C-reactive protein, *ULN* upper limit of normal

**Fig. 1:**
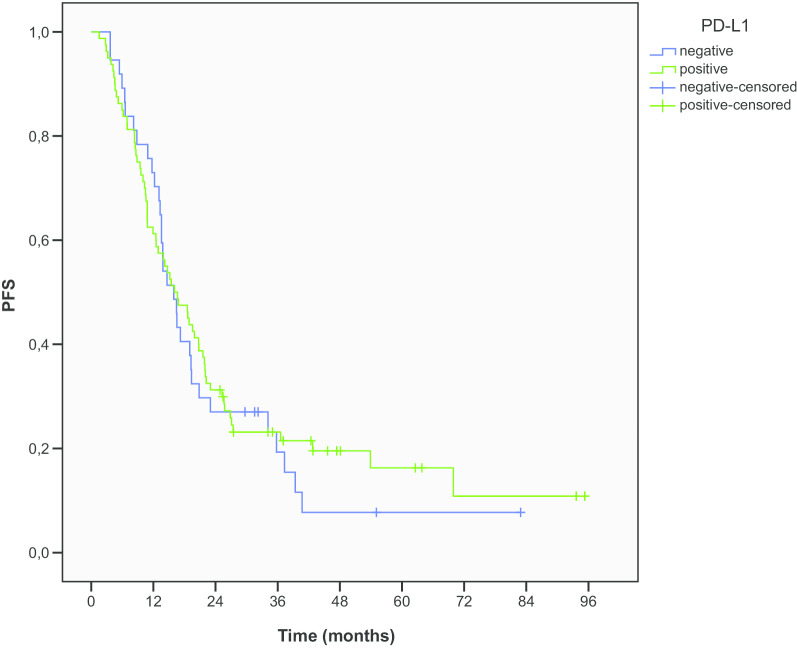
PFS according to PD-L1 expression

**Fig. 2:**
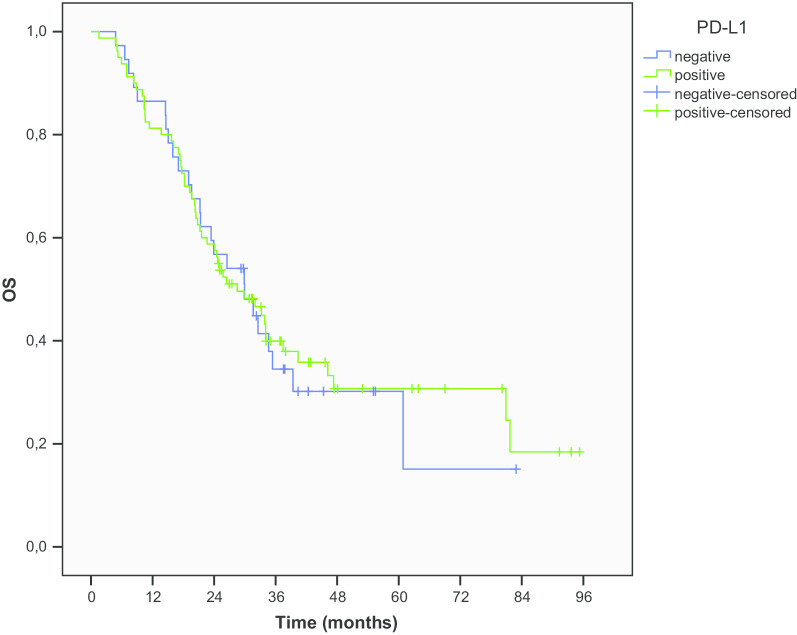
OS according to PD-L1 expression

Median PFS (mPFS) was 15.9 months in PD-L1 negative patients and 16.1 months in patients with PD-L1 ≥ 1% expression (*p* = 0.696) (Fig. 1). Median OS (mOS) in PD-L1 negative patients was 29.9 months compared to 28.5 months in patients with PD-L1 ≥ 1% expression (*p* = 0.888) (Fig. 2). The OS rates at 1, 2 and 5 years were 86.5%, 56.8% and 30.2% for PD-L1 negative patients and 81.3%, 58.7% and 29.1% for patients with PD-L1 ≥ 1% expression, respectively. At the time of last evaluation in January 2020, 43 patients were alive, 12 (32.4%) PD-L1 negative and 27 (33.7%) with PD-L1 ≥ 1% expression.

The percentage of patients with PD-L1 tumor cells expression of more than 50% was 29.9%. There was no association between ≥ 50% PD-L1 expression and age, gender, histology, smoking status and ECOG PS (Table 2). Regarding the stage, between the subgroups of PD-L1 ≥ 50% and less, the unbalance was noticed in the subgroups of the stage III but the higher PD-L1 was not associated with higher stage (p = 0.0230). There was no difference in mOS in patients with ≥ 50% PD-L1 expression with 29.8 months compared to 29.9 months in those with < 50% or no PD-L1 expression (p = 0.694).

**Table 2:** Clinical characteristics of patients regarding PD-L1 expression

Characteristics	All, n	PD-L1 < 50%, n	(%)	PD-L1 ≥ 50, n	(%)	p
All patients	117	82	70.1	35	29.9	
Age (years)						0.236
Median	61.0	60.5		64		
Range	47–77	47–77		50–75		
Gender						0.117
Female	38	23	28	15	42.9	
Male	79	59	72	20	57.1	
Histology						0.201
Squamous	71	48	58.5	23	65.7	
Adeno	39	27	32.9	12	34.3	
Other	7	7	8.5	0	0	
Smoking status						0.702
Current	67	49	60.5	18	52.9	
Former	44	29	35.8	15	44.2	
Never	4	3	3.7	1	2.9	
Stage						0.0230
IIIA	30	17	20.7	13	37.1	
IIIB	75	59	72.0	16	45.7	
IIIC	12	6	7.3	6	17.1	
ECOG PS						0.625
0	58	41	50.0	17	48.6	
1	57	39	47.6	18	51.4	
2	2	2	2.4	0	0	
RT dose (Gy**)**						0.639
Median		60		60		
Range		54–66		54–66		
ChT (number of cycles)						0.666
Median		3		3		
Range		1–5		1–5		
CRP after ChT/RT						0.447
≤ 2 × ULN	74	51	62.2	23	69.7	
> 2 × ULN	41	31	37.8	10	30.3	

*ChT* chemotherapy, *ECOG PS* Eastern Cooperative Oncology Group performance status, *n* number, *RT* radiotherapy, *CRP* C-reactive protein, *ULN* upper limit of normal

**Fig. 3:**
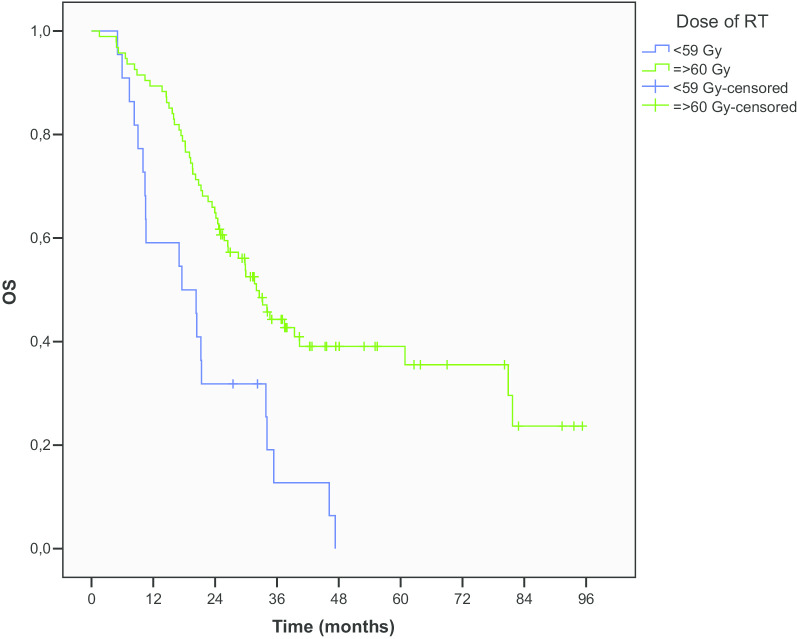
OS according to RT dose

According completion of the treatment no difference was revealed regarding the number of ChT cycles. However, the total dose of 60 Gy or more was associated with significantly better mOS (32 vs. 17.5 months, p < 0.001, HR 0.38). OS rates at 1, 2 and 5 years were 88.4%, 64.2% and 38.7% for doses ≥ 60 Gy compared to 59.1%, 31.8% and 0% for lower doses (Fig. 3).

In further evaluation, we analyzed pre-treatment and post-treatment inflammatory markers, NLR and serum CRP as the marker of systemic inflammatory response regarding outcome of patients. With cut-off 5 for pretreatment NLR we found limited association between high NLR level and poorer OS (p = 0.059) as for increased CRP level more than two times of normal (p = 0.063). After the completion of treatment increased CRP level more than two times of normal was associated with significantly shorter mPFS (12.5 vs. 19.5 months, p = 0.009) and mOS (20.3 vs. 34.0 months, p = 0.005) compared to lower levels of CRP in univariate analysis, but this was not confirmed in the multivariate analysis.

**Fig. 4:**
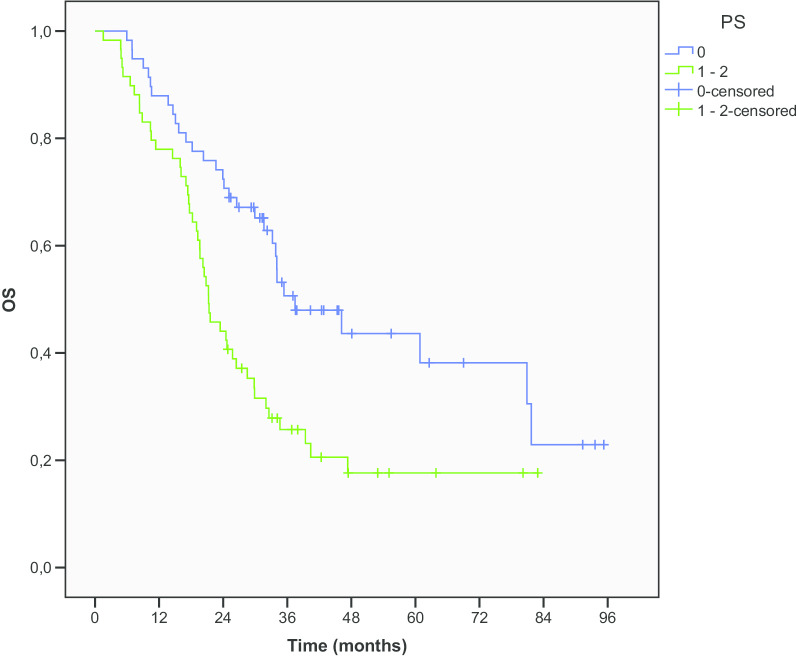
OS according to PS

Regarding clinical characteristics, our analysis revealed significant better mOS in patients with ECOG PS 0 compared to PS 1–2 (37.4 vs. 21.3 months, p = 0.002, HR 1.87) (Fig. 4).

Multivariate analysis demonstrated that the PS and RT dose were independent and significant predictive factors of OS (Table 3).

**Table 3:** Univariate and multivariate analyses of clinicopathologic factors according to PFS and OS

Factor	n	PFS			OS		
		Median (months)	Univariate p value	Multivariate p value (95% CI)	Median (months)	Univariate p value	Multivariate p value (95% CI)
Age							
≤ 61	59	15.9	0.549		28.5	0.533	
> 61	58	16.1			29.9		
Sex							
Male	79	15.9	0.908		24.1	0.267	
Female	38	16.5			32.0		
Stage							
IIIA	30	19.5	0.306		34.6	0.269	
IIIB	75	13.9			26.5		
Histology							
Squamous	71	15.9	0.740		24.1	0.190	
Adeno	39	16.7			34.0		
ECOG PS							
0	58	18.6	**0.078**	0.273	37.4	**0.002**	**0.013**
1–2	59	14.2		(0.830–1.936)	21.3		**(1.145–3.066)**
PD-L1							
Negative	37	15.9	0.696		29.9	0.830	
Positive	80	16.1			28.5		
PD-L1							
< 50%	82	14.7	0.362		29.9	0.689	
≥ 50%	35	19.4			29.8		
RT dose							
< 60 Gy	22	10.6	**0.014**	**0.019**	17.5	** < 0.001**	**0.001**
≥ 60 Gy	95	18.6		(0.321–0.905)	32.0		**(0.218–0.667)**
NLR pre							
< 5	87	17.2	0.532		34.0	**0.059**	0.087
≥ 5	23	13.8			21.4		(0.933–2.810)
NLR post							
< 5	48	19.5	0.408		34.6	0.120	
≥ 5	65	13.5			24.1		
CRP pre							
≤ 10	54	20.7	**0.093**	0.217	37.5	**0.063**	0.105
> 10	56	13.3		(0.855–1.990)	24.1		(0.919–2.416)
CRP post							
≤ 10	74	19.5	**0.009**	0.221	34.0	**0.005**	0.250
> 10	41	12.5		(0.842–2.099)	20.3		(0.808–2.265)

Results with values of p < 0.2 in univariate analysis were calculated in multivariate analysis. A p-value less than 0.05 was considered statistically significant

*ECOG PS* Eastern Cooperative Oncology Group performance status, *n* number, *PFS* progression free survival, *OS* overall survival, *RT* radiotherapy, *NLR* neutrophil lymphocyte ratio, *CRP* C reactive protein, *pre* pre-treatment, *post* post-treatment

## Discussion

The objective of present analysis was the confirmation of our previously published data about prognostic value of PD-L1 expression on survival of patients with unresectable NSCLC in stage III that were treated with definitive combined RT and ChT [11]. In present retrospective analysis, we found no difference between PFS and OS of patients with NSCLC neither in group of ≥ 1% nor in ≥ 50% PD-L1 expression. Our decision to analyze two groups, regarding PD-L1 cut-off value ≥ 1% or ≥ 50% was based on the results of two studies that have confirmed benefit of treatment with checkpoint inhibitors in first-line setting for patients with metastatic NSCLC. [17, 18]. In addition, for inoperable stage III NSCLC patients, the results of the Pacific study have demonstrated significantly higher PFS and OS with maintenance durvalumab after successful treatment with concomitant ChT and RT. In Europe, this combination became a new standard of care for inoperable stage III NSCLC with PD-L1 expression ≥ 1% [1–3].

The proportion of patients in our present analysis with 68.4% of patients with ≥ 1% and 29.9% with ≥ 50% PD-L1 expression is comparable to data from the literature. In a large, consecutive study of PD-L1 expression in all stages of NSCLC in Denmark, 791 patients were included [13]. Of those, 63% had PD-L1 ≥ 1% positive cells and 30% had PD-L1 ≥ 50% positive cells. Of 160 patients in stage III, the proportion was similar with 65% for PD-L1 ≥ 1% and 28% for PD-L1 ≥ 50% positive cells. However, the expression of PD-L1 in the largest global multicenter Express study with stage IIIB/IV NSCLC patients was lower than in our analysis [19]. Of 2368 patients from 45 centers across 18 countries on different continents, 52% had PD-L1 tumor proportion score ≥ 1% and 22% had score ≥ 50%. Of all cases, 831 were included from Europe and the prevalence of the PD-L1 expression was the same as for the entire group. Limitation of that study is retrospective nature of analysis from archival samples that might not be collected consecutively in all cases. In addition, cytological specimens were not included in the analysis. Recently, Gelatti et al. reported lower prevalence in Brazilian cohort with 43.46% of NSCLC patients with score ≥ 1% of PD-L1 expression and 17.83 ≥ 50% [20]. In all four studies, PD-L1 expression was evaluated by staining with Dako PD-L1 IHC 22C3 pharmDx kit. PD-L1 expression in our specimens was analyzed with the Ventana PD-L1 SP263 assay. However, inter-assay concordance of PD-L1 expression is reported to be high between standardized assays 22C3, 28-8 and SP263, while assay SP142 shows lower expression on tumor cells [21].

Our analysis of prognostic value of PD-L1 expression in consecutive homogenous group of 117 patients with stage III unresectable NSCLC, treated with combination of ChT and RT is the largest one published until now. Two other studies have investigated prognostic value of PD-L1 in homogenous group of unresectable patient in stage III NSCLC. In the first, Takito et al. have demonstrated that PD-L1 expression was not correlated with PFS and OS in 74 consecutive patients in stage III NSCLC who had received concurrent chemoradiotherapy. [22] Positive tumor PD-L1 staining was present in 74% of the patients, similar as in our analysis, but the cut-off value for negative tumors was set at 5%. Their examination also included the immunohistochemically evaluation of CD8+ TIL density and they demonstrated in univariate and multivariate analysis that it was independent and significant predictive factor for PFS and OS. Sub-analysis revealed that the patients with lack of PD-L1 expression in combination with high CD8+ TIL density had better survival. In the second analysis, recently published by Gennen et al., 31 patients with locally advanced (90.3%) and metastatic (9.7%) NSCLC were included. [23] PD-L1 expression less than 1% on tumor cells was associated with improved OS, PFS and local control in patients treated with concurrent ChT and RT. In that study assessment of CD8+ TIL density was also performed and according to both features, all patients were stratified to four different types of tumor immune microenvironment. The shortest OS had patients with PD-L1 expression and low CD8+ TIL density, which was in accordance to the previous mentioned study, but the longest OS had patients with negative PD-L1 expression and low CD8+ TIL density. However, limitation of both retrospective studies should be mentioned. Both cohorts were small, the threshold for positivity was not clearly defined and comparable, and various antibodies were used. Most importantly, the tumor samples size obtained from bronchoscopy might be too small to evaluate TILs density adequately.

More extensive data regarding prognostic value of PD-L1 expression are available from retrospective reports, systematic reviews and meta-analysis in resected NSCLC patient. Few of the analysis that included large numbers tissue samples from resected NSCLC patients (289, 705, 1016 and 982 patients) reported no impact of PD-L1 expression on PFS and OS [24–27]. On the contrary, some reported significant impact of PD-L1 expression on prognosis. Recently, results of a single institutional study of clinicopathological features regarding PD-L1 with a large number of patients of the Polish population were published [28]. A cohort of 866 patients with stage I to IV NSCLC that underwent surgery from January 2007 to December 2011 reveled association of higher PD-L1 expression with higher grade of malignancy, higher N stage and higher stages of NSCLC. Additionally, adenocarcinoma patients with high PD-L1 expression (≥ 50%) had shorter survival compared to those with low PD-L1 expression (p = 0.0332) but this difference was lost when patients were divided into two groups with or without PD-L1 expression. Other researchers reported PD-L1 expression as a poor prognostic factor for patients with NSCLC, mostly in Asian patient cohorts. [29–35] On the contrary, three studies of Western and Australian patient cohorts found PD-L1 expression as a favorable prognostic factor [36–38]. In the largest Western cohort of surgically resected stage I–III NSCLC cases in European Thoracic Oncology Platform (ETOP) Lungscape Project, 2008 evaluable cases were assessed regarding PD-L1 expression [36]. PD-L1 positivity prevalence of 1% and 50% cut-off was 43.4% and 16.6%, respectively, but was significantly more frequent in higher stages. PD-L1 positivity was associated with better prognosis for non-metastatic NSCLC patients and for adenocarcinoma patients, but no effect was found for the squamous cell carcinoma. Cooper et al. reported that high PD-L1 expression was associated with younger patient age and high tumor grade and in multivariate analysis, those patients had significantly longer overall survival [37].

Despite conflicting data about prognostic value of PD-L1 expression, results regarding higher PD-L1 expression in patients with higher stage of NSCLC are more convincing. In a Denmark study, stage was reported as the most important predictor of PD-L1 expression with higher stages having higher prevalence of PD-L1 expression and odds ratio of 0.31 for stage I vs. stage IV [13]. Similarly, in a study of Wang et al. the stage of NSCLC in 483 patients was significantly associated with PD-L1 positivity [12]. PD-L1 expression ≥ 50% was 2.3-fold higher in metastatic NSCLC compared to early and locally advanced NSCLC. In a large cohort study of Sun et al., 1070 patients with NSCLC surgically resected between 2001 and 2010 of a single institution in Korea, were included [39]. PD-L1 expression in a multivariate analysis was more common in squamous cell carcinoma and higher stage. The prevalence of high as well as low PD-L1 expression versus no expression was statistically higher in patients with more advanced NSCLC than in early-stage disease. In our analysis, we could not confirm the difference regarding higher PD-L1 expression in higher stages. Probably, the number of patients was too small to detect difference between subgroups of stage III. Additionally, most other studies reported the differences between stages I–IV.

In the light of inconsistent prognostic value of PD-L1 expression in NSCLC patients, it is likely that the prognostic significance relates to the overall balance of the host anti-tumor immune response and tumor-mediated immunosuppression. PD-L1 expression might be only one of the players in this equilibrium. We should look into the broader picture because more molecular markers of tumor microenvironment and the host immune response might be considered as a predictor of NSCLC patient’s prognosis. There is evidence that expression of PD-L1 is an adaptive mechanism and may be a marker of tumor response to the host immunity rather than intrinsical tumor marker [40]. This might explain the findings of those studies that associate prevalence of PD-L1 expression with higher stages [12, 13, 28, 39]. The host immune response to tumor growth can also include inflammation and an increase in serum CRP. Systemic inflammation and elevated CRP is linked to poor outcome in NSCLC patients [41, 42]. Serum CRP is a sensitive marker of acute-phase systemic inflammation. Elevated CRP as a response to elevated cytokine levels after inflammatory stimulus is mainly produced by hepatocytes [43]. On the other hands, cancer cells have been shown to express CRP and produce various cytokines [44]. Among them, IL-6 was reported to be the only inflammatory cytokine independently associated with serum CRP concentrations in patients with advanced-stage NSCLC [44]. The data suggests serum CRP as a useful substitute marker of IL-6 activity in NSCLC patients. IL-6 was reported to importantly contribute in cancer growth and progression by activating IL6R-JAK-STAT3 signaling pathway [45]. Over-activation of STAT3 pathway increases cell-cycle progression, tumor invasion, angiogenesis, metastatic spread and suppress apoptosis [42]. Data shows that STAT-3 activation as a mediator of inflammation plays an important role in tumorigenesis as well [46, 47]. In addition, there are data that STAT-3 has a pivotal role in regulating PD‐L1 expression, triggered via IL‐6 and IL‐10 [48]. Systemic inflammatory response in cancer involves many organ systems, and usual clinical laboratory measurements besides acute phase inflammatory proteins, it also include circulating white cells [14, 15, 49]. In particular, blood cell counts such as neutrophil, lymphocyte and platelet counts, NLR, platelet lymphocyte ratio and Glasgow Prognostic Score based on serum CRP and albumin have been reported to have prognostic value [14, 15, 50–52]. However, there has been no reference cut-off point for NLR that could be used for clinical use till now. The range of cut-off points reported to have prognostic impact varied from 2.5 to 5 in different studies. [15] In our present analysis, NLR was not associated with survival, but we found that patients after the completion of RT had longer mOS when CRP is under 10 (2 × ULN) compared to patients with higher values (34.0 vs. 20.3 months, p = 0.005). However, the difference was not significant in multivariate analysis.

As expected, RT dose ≥ 60 Gy and good PS were independent and significant predictive factors of better mOS in our multivariate analysis [53–56].

One important limitation of our present analysis is its retrospective design. Only patients with stage III unresectable NSCLC that completed radiotherapy with 54 Gy or more were included. We have no data of patients that never completed intended radical treatment due to progression or adverse events and might influenced the results of this analysis. In addition, the archival samples acquired from 2012 to 2017, were analyzed in 2019 and PD-L1 expression could be compromised over the years of storage. Moreover, possible important issue was small biopsy samples and intra-tumoral heterogeneity of PD-L1 expression.

## Conclusion

In our cohort of 117 consecutive unresectable NSCLC patients in stage III treated with chemoradiotherapy PD-L1 expression from biopsy samples taken before therapy had no prognostic value regarding PFS and OS, neither comparing the groups of patients with more or less than 1% PD-L1 expression or groups with more or less than 50%. Patients with PS 0 and those who received more than 60 Gy of radiation dose had significantly better OS.

## Data Availability

The datasets used and analysed during the current study are available from the corresponding author on reasonable request.
